# Why Do Communities Recover Differently after Socio-Natural Disasters? Pathways to Comprehensive Success of Recovery Projects Based on Bam’s (Iran) Neighborhoods’ Perspective

**DOI:** 10.3390/ijerph19020678

**Published:** 2022-01-07

**Authors:** Homa Bahmani, Wei Zhang

**Affiliations:** Department of Construction Management, School of Civil Engineering and Hydraulic, Huazhong University of Science and Technology, Wuhan 430074, China; homabahmani@hust.edu.cn

**Keywords:** disaster recovery projects, successful recovery, Bam earthquake, fuzzy-set qualitative comparative analysis, community recovery

## Abstract

Although recent studies have provided explanations for the causes of success and failure in recovery projects following socio-natural disasters, there is a need for a concise understanding of how different combinations of factors may contribute to recovery failure or success. In this study, to examine the community recovery pathways after the 2003 Bam earthquake, we conducted a fuzzy-set qualitative comparative analysis of 11 neighborhoods in Bam city and Baravat (the neighborhoods represent the division of the areas damaged by the earthquake, as presented by local government). The success of socio-natural disaster recovery projects is presented in three pathways in which the absence or presence of public engagement had a significant influence on the results. The results indicate that a recovery project should respond to the needs of the project within the continuous lifecycle of the project. Additionally, on the one hand, public participation and prompt rescue operations have a significant effect on project success. On the other hand, neglecting the needs of people and the area’s climate in housing design have led to project failure. It is expected that the findings from this study can be used to develop strategies for empowering people in recovery projects and to develop housing guidelines that respect residents’ needs while focusing on on-time and sufficient rescue processes. However, care should be taken when applying the present findings in practice, because every socio-natural disaster is unique and requires careful consideration of complex sets of features.

## 1. Introduction

According to the Center for Research on the Epidemiology of Disasters (CRED), a disaster is “a situation or event that overwhelms local capacity, necessitating a request at the national or international level for external assistance; an unforeseen and often sudden event that causes great damage, destruction, and human suffering” [1]. However, it is necessary to differentiate between hazards and disasters, since the latter is the result of the former occurring in a vulnerable environment [2]. To address the last mentioned concern, in this study, we use “socio-natural disasters”, which differentiates disasters triggered by natural hazards from the other disasters [3,4].

According to the World Bank reports, 50% of Socio-Natural Disaster Recovery (SNDR) projects fail to achieve their projects goals [5]. There are extensive studies in the literature that have investigated the reasons for SNDR projects’ failure. A shortage of integration among different sectors, financial limitations, communication and coordination deficiencies, inadequate resource procurement, ineffective designs, transportation difficulties, corruption, and delay are some of the bottlenecks that obstruct successfully conducting SNDR projects [6]. Additionally, due to the engagement of multiple stakeholders [7], the likelihood of conflicts in SNDR projects is higher, influencing coordination and communication among beneficiaries [8].

The success or failure of SNDR projects can be evaluated through different methods. While some researchers have focused on improving the built environment, many studies have suggested several dimensions for assessing recovery success. Overemphasis on one recovery dimension may affect the balance among recovery goals [9]. It has been suggested that recovery assessment covers several dimensions, from physical progress in construction to economic, social, and mental welfare improvement [10,11]. An evaluation of the recovery process was presented by *Wu*, who focused on the tangible results achieved as well as social progress, emergency responses, and decision-making [8]. Moreover, evaluating the success of SNDR projects depends on stakeholders’ perspectives, who have varied needs that differ from those of local citizens and governments [12].

Critical Success Factors (CSFs) that influence the success of a project have been widely discussed. While recent studies have widely assessed the effectual factors of successful SNDR projects, there is still limited research that has examined the relationships among CSFs and a specific group of outcomes. Numerous CSFs may affect recovery outcomes; however, the factors that influence recovery success based on perspectives within a community can be listed as follows: effective decision-making and coordination [6,13,14,15,16], integration of community recovery planning processes [13], public participation [13,14,15,16], public awareness [13], effectively administered and sufficient financial acquisition [6,13], resilient rebuilding [13], available recovery resources [13], and consideration of people’s needs and culture in planning [6,14,17]. Although the positive impact of those factors has received widespread acknowledgement, there is still disagreement about the effects of some parameters. Several studies have focused on maximizing people participation; however, controlling the people’s engagement based on the policies has offered deliberate public engagement in SNDR projects [6,18]. A study conducted by Kermanshachi et al. systematically categorized post-disaster recovery barriers and analyzed how they interacted with each other. *Jordan* also defined recovery pathways in the communities affected by the 2004 Indian Ocean tsunami [19]. Nevertheless, this topic needs further investigation as different types of disasters in other locations may demonstrate dissimilar results.

To evaluate the combination of factors that lead to successful achievement of project goals across case studies, the most commonly used method is Qualitative Comparative Analysis (QCA), which *Charles Ragin* introduced in 1987 [20]. This method, known as the third way of data analysis, bridges the gap between qualitative and quantitative analysis by better understanding the complex causes of success and failure. In addition, the logic behind this method differs from traditional qualitative and quantitative techniques, as it benefits from set theory and Boolean algebra [21].

In this study, we used the QCA method to analyze data collected by structured interviews with household members and observations conducted in the Bam area, Iran, to evaluate how combinations of effectual parameters might lead to successful recovery projects after the 2003 Bam earthquake. The combinations of factors leading to success or failure of defined outcomes were assessed using fuzzy-set Qualitative Comparative Analysis (fsQCA). Finally, a holistic strategy leading to a successful recovery project following the Bam community’s perspective is defined by comparing success and failure pathways. The recognized ways for both failure and success can help governments, decision-makers, and planners to optimize the recovery process, and therefore, achieve the desired goals within a shorter time period and with less waste of resources.

## 2. Materials and Methods

### 2.1. Research Setting

Bam is a historical city located in the southern Iranian Province of Kerman. Before the earthquake, Bam was categorized as an economically poor region, where 25% of people’s careers was in agriculture, especially palm grove production. Husbandry was the other common career in Bam. Tourism in Bam was also booming because of the presence of the world cultural heritage site: the largest adobe structure, the ancient citadel (Arg-e-Bam) [22].

**Figure 1:**
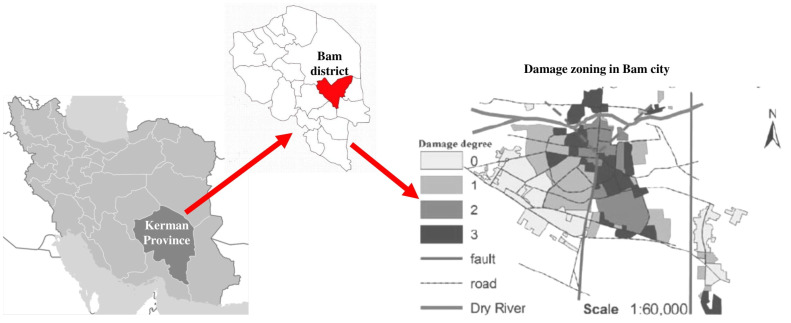
Bam earthquake location and damage zoning map.

On 26 December 2003, a 6.7 magnitude earthquake hit Bam. A total of 142,000 people were affected, among whom more than 26,000 people died, approximately 30,000 people were injured, and 100,000 people were left homeless [9]. The Bam earthquake happened on a weekend at midnight and caused severe casualties, as people had limited time to escape [22]. The quake caused the destruction of 85% of the homes and city infrastructures as well as many commercial centers in the district [23]. The earthquake, with an epicenter depth of 10 km, resulted in minor damage in nearby villages; the most severely affected areas were located 15 km away from the epicenter [22]. Figure 1 shows the location of the Bam district in Iran and graphically shows the degree of damage caused by the earthquake in 2003.

### 2.2. Data Collection

#### 2.2.1. Identification of Conditions and Outcomes

**Figure 2:**
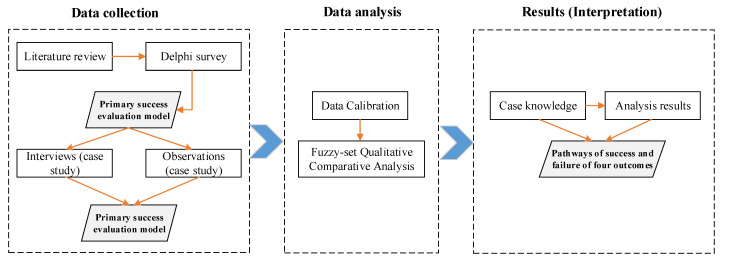
Research steps. Rectangles stand for research tools, while the parallelograms signify research milestones.

Generally, a QCA defines which factors/combination of factors might affect the results [21]. The application of QCA in SNDR projects enables the researchers to identify where, when, and under what circumstances projects can be conducted successfully [21]. However, three necessary steps should be completed before the application of QCA. First, the outcome(s) must be determined. The outcome is the objective or result (desirable or undesirable) of the procedure. Second, the conditions, which are the characteristics that affect the outcome(s), must be defined. Finally, the numerical values of the chosen outcomes and conditions are determined by constructing truth tables based on data collected from the selected cases [24]. The research steps of this study are graphically illustrated in Figure 2.

First, the factors that affect the failure or success based on the public perspective of SNDR projects were listed. The conditions were identified through an in-depth literature review. Then, experts’ opinions were collected through a Delphi survey to refine the extracted factors. Ten panelists were selected from well-known researchers in disaster recovery who had a PhD degree in the relevant fields, at least one academic journal paper about SNDR projects, and more than three years of experience in disaster-related fields.

To comprehensively assess the public perspective on recovery outcomes, in this study, we divided outcomes into three groups, namely, resilient society, sustainable and resilient built environment, and resilient economy. The conditions attempted to cover the continuous life-cycle of SNDR projects (short-term, mid-term, and long-term periods), and therefore, we applied the time-based life-cycle proposed by *Bahmani and Zhang* [25]. By applying the continuous life-cycle for SNDR projects and systematic categorization, we ensured that a wide range of factors were considered in the proposed success evaluation model.

**Figure 3:**
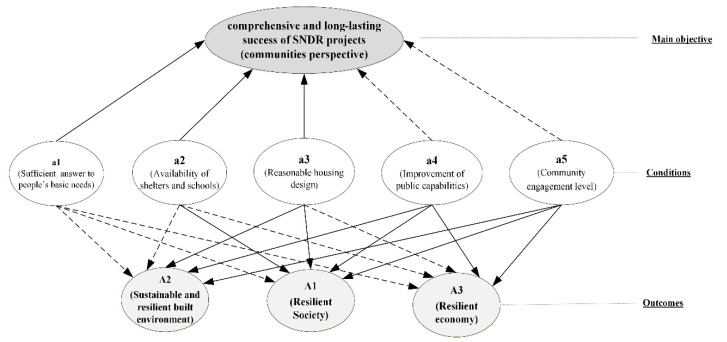
Success evaluation model for SNDR projects per community perspective. Dashed lines indicate the presence and absence of conditions that may affect the connected outcome, while solid lines represent the presence of conditions that affect the subsequent outcome.

Moreover, the established conditions and outcomes were revised based on knowledge of the neighborhoods. The domain factors such as evacuation facilities [26], food availability [27,28], and equity among the populations [29,30,31] were removed from the analysis, as they did not vary across the neighborhood [24]. We placed the recognized factors in a tree-shaped categorization to reduce the number of the parameters and modeling complexity [20]. The conditions, outcomes, and references are listed in Table 1 and Table 2. After the mean values of the outcomes were obtained from the Delphi survey, shown in Table 1, the final score of the ultimate goal was calculated through a weighted average of the mean scores for outcomes A1, A2, and A3, giving twice the weight to outcome A3. Figure 3 illustrates the most important components and their interactions in the model. We considered the presence of the conditions leading to the subsequent outcome unless there was no strong theoretical link between the outcome and conditions.

#### 2.2.2. Neighborhood Assessment

**Figure 4:**
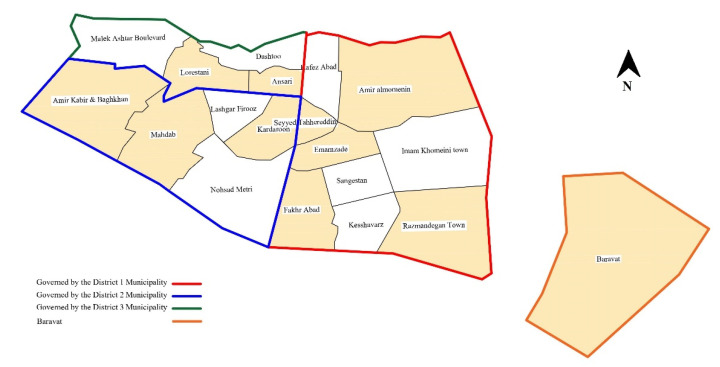
Selected neighborhoods in Bam and Baravat.

Next, the community-based success evaluation model for SNDR projects was applied to neighborhoods affected by the 2003 Bam earthquake. The data were collected through structured interviews with households in 11 neighborhoods in Bam city and Baravat. Since Bam was reconstructed by dividing it into several districts assisted by different provinces, the neighborhoods were selected according to the damage zoning map after the earthquake and we attempted to cover various sections governed by each of three municipalities in Bam. Moreover, Baravat was chosen because of its proximity to Bam city (almost 10 km) and the high level of destruction caused by the earthquake. Figure 4 shows the chosen neighborhoods (colored districts) where the public survey was conducted.

Two trained interviewers conducted the interviews in chosen neighborhoods during August 2021. The interviewers were provided with a detailed guide of conducting public interviews (general requirement to interact with people), choosing interviewees, and research goals and backgrounds. The pieces of training were both in the form of written materials and online discussions. We have kept close contact with them during the fieldwork and held online meetings once a day. In case of any difficulty during the fieldwork, they immediately contacted us and asked for further information. The main criterion for choosing the interviewees was their living duration in the damaged area; interviewees must have lived in Bam/Baravat before the earthquake. Moreover, the interviewers selected only one person from each household to avoid redundant data. The fieldwork lasted for five days and resulted in 122 interviews with members of the public, among which 67% of interviewees were male, 48% of the interviewees were between 31–40 years old, and 45% of interviewees had been working in government-related organizations/agencies. An integrated online form was utilized to collect the responses. The interview responses were recorded in Farsi and then stored. The interviewers also observed the neighborhoods, city facilities, urban development, and emergency preparedness in the selected neighborhoods. Finally, the non-weighted average of each neighborhood’s responses represented the neighborhood’s score. In the case of significant differences among the collected responses in one neighborhood, the researchers revised the data based on their observations and secondary documents.

### 2.3. Data Analysis

#### 2.3.1. Calibration Rubrics

Once the data were collected, the researchers began the data analysis by initiating data calibration; all the collected data were indirectly calibrated based on set theory and data matrices were constructed. The questions’ answers were calibrated using fuzzy-set logic along with general knowledge the researchers gained about the neighborhoods. Since most factors consist of several subfactors, calculation formulas depend on the role and importance of the factors. If all the subfactors are equally important, the average score of the subfactors is calculated. For example, for outcome A1, i.e., resilient society, there were four subfactors that were equally important. Furthermore, the total score for this outcome was a non-weighted average of the four subfactors. If previous research and knowledge of neighborhoods emphasized one subfactor, the average score was calculated considering weights. For example, reasonable housing design emphasizes consideration of the culture and local needs. Therefore, the average score was calculated, giving twice the weight to housing based on the culture of the damaged area and the needs of the people who were affected and one weight was given to climate design. If the researchers recognized all the factors must be present in the set, the minimum score was calculated. For example, “road improvement” is a sub-outcome of outcome A2, which consists of several subfactors. Since the researchers recognized that all the factors must be present to have better roads, the minimum score of the subfactors formed the total score of this sub-outcome. The maximum score in the calibration table indicates that the presence of one of the parameters is enough for the neighborhood to be considered in the set. An example of the calibration rules is given in Table 3 (the complete calibration rubrics are presented in the Appendix A, Table A1 and Table A2).

#### 2.3.2. Fuzzy-Set Qualitative Comparative Analysis (fsQCA)

After the data were calibrated, truth tables containing the scores for each condition and outcome for each neighborhood were constructed. The “necessity” and “sufficiency” of individual conditions can also help the researchers to input conditions that are logically connected to the outcomes for the next phase. While necessity shows the degree to which an outcome is a subset of causal conditions, sufficiency reveals the degree to which the causal condition is a subset of the outcome(s) [56,57]. Next, the truth table was accessed through logical minimization illustrating the pathways (combinations of condition(s) leading to a defined outcome). “Consistency” and “coverage” are two measurements used to evaluate pathways recognized as “parameters of the fit.” The degree to which the given cases of the causal conditions present outcomes can be calculated by consistency. Coverage indicates the degree to which the pathway was observed among the cases. Usually, the researchers prefer a consistency of at least 0.8 among the recognized pathways [20]. In this study, the ultimate outcome, the comprehensive and long-lasting success of SNDR projects per a community’s perspective, was evaluated by calculating the average scores of the major outcomes, i.e., A1, A2, and A3, given twice the weight of A3, following the Delphi results. Table 4 shows the neighborhoods’ scores for the model’s outcomes and conditions.

In this study, we used fsQCA software to code the truth tables and run the logical minimization formula. Since an intermediate solution applies the more likely assumptions and provides a researcher with the freedom to choose the absence or presence of the conditions leading to the outcome, the results of the intermediate solution were obtained using the Quine–McCluskey algorithm. Following the QCA logic, simply negating the success pathways may not create pathways for failure. Therefore, we also modeled the negation of each outcome separately, resulting in eight models with four outcomes and their negations.

## 3. Identification and Interpretation of Success and Failure Pathways of the Outcomes

In this section, we provide a detailed evaluation of the research findings, recognizing the causal combinations of conditions that lead to the success or failure of outcomes and the ultimate objective shown in Figure 3. Conditions that did not show a strong theoretical link to the outcomes were considered to be simultaneously present and absent. The process started with the calculation of necessity and sufficiency scores of individual conditions. Note that the order of the conditions in the pathways does not imply the sequence of their occurrence. In each path, the conditions must be present or absent (shown by ~) to gain the outcome. The outcome can be achieved through any of the pathways. The calculation procedure of QCA method was presented in Appendix B.

### 3.1. Resilient Society (A1)

**Figure 5:**
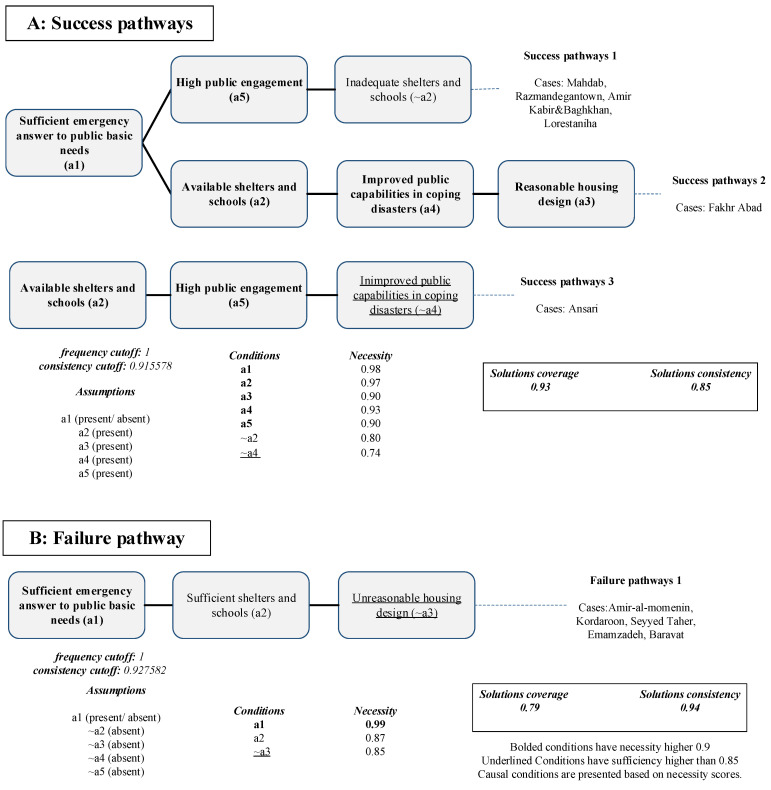
Success and failure pathways for outcome A1.

The three recognized success pathways for a resilient society appear in all the neighborhoods identified as successful and show an overall consistency of 0.85 and coverage of 0.93. The recognized pathways for a resilient society are presented in Figure 5. It can be observed that sufficient emergency response (condition a1) is the condition that appears in most successful neighborhoods (four of five neighborhoods). This finding is in line with many other studies, suggesting that on-time rescue and adequate services during the emergency phase can affect the resiliency of communities [58]. Moreover, public engagement (condition a5) appears in two pathways that account for four successful neighborhoods. Communities with a high level of public engagement are expected to achieve a resilient society by building social connections, achieving faster psychological recovery, and experiencing higher recovery satisfaction [3,59].

Interestingly, the availability of shelters and schools (condition a2) appeared in all the solutions; however, it was present in the second and third pathways but absent in the first one. Mahdab, Razmandegan town, Amir Kabor and Baghkhan, and Lorestaniha were identified as resilient neighborhoods, although they have a shortage of safe shelters and schools during the transitional phase (~a2). However, the rescue process in those neighborhoods was highly satisfactory (condition a1), as residents had acceptable access to sanitation facilities and the average start time of the rescue was shorter than in the other neighborhoods. In addition, public engagement (condition a5) was sufficient in those neighborhoods, contributing to the success of outcome A1.

In addition, the Fakhr Abad neighborhood presented the second pathway, which revealed that a resilient society can be achieved if the community receives adequate assistance in the emergency, transitional, mid-term, and long-term stages of recovery. It is quite surprising that reasonable housing design was only presented in the second pathway. Although it gained high necessity, it did not reach a high sufficiency score (necessity = 0.90 and sufficiency = 0.84). However, its absence is significantly sufficient to fail to achieve a resilient society, although not necessarily leading to the failure (necessity = 0.85 and, sufficiency = 0.91). The failure pathway of outcome A1, presented in Figure 4 was present in all unsuccessful neighborhoods. Sufficient emergency response to basic public needs (conditiona1) is the only necessary condition for failure whose combination with sufficient shelters and schools (condition a2) and unreasonable housing design (~a3) resulted in failure in outcome A1.

### 3.2. Sustainable and Resilient Built Environment (A2)

**Figure 6:**
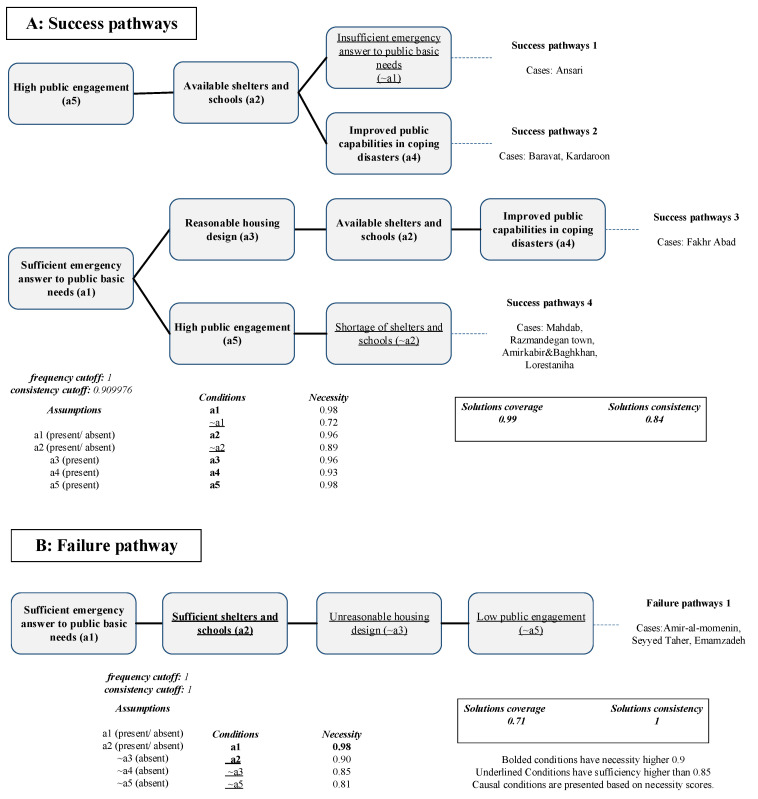
Success and failure pathways for outcome A2.

Four pathways presented in eight neighborhoods were identified to achieve a sustainable and resilient built environment. The overall consistency and coverage score of the solution was 0.84 and 0.99, respectively. The success and failure pathways are presented in Figure 6.

High public engagement (condition a5), which appeared in three pathways and was present in seven neighborhoods, is the condition most strongly linked to a successful sustainable and resilient built environment. This finding aligns with the findings by previous researchers, who stated that city planning should consider the real needs of people by engaging senior citizens and civic groups in disaster planning scenarios and organizing frequent neighborhood events [26]. The Ansari neighborhood presented the first path and had the highest score in community participation, as well as a remarkable public information sharing score. Although informing people is at the third level in the ladder of community participation [35], it can positively affect a sustainable and resilient built environment. Finally, people’s participation in housing was a critical factor in Bam’s neighborhoods. Most households in the Ansari neighborhood were in charge of housing, acting as managers of their houses’ construction, and working under the government’s guidance. High community participation in the reconstruction phase can result in a sense of neighborhood ownership, resulting in high satisfaction [3,60].

According to the interviews and the damage zoning maps, the Razmandegan neighborhood, located at the south corner of the city, did not have many residents at the time of the earthquake. Since it was further from the earthquake fault, it also experienced less damaged. However, after the quake, nearby villagers who had experienced less damage from the earthquake rushed to this neighborhood, causing insufficient available shelters. On the contrary, the Ansari neighborhood located near the city center was ranked as a second-level destroyed neighborhood receiving inadequate emergency response. However, the residents in both neighborhoods positively addressed the city’s changes after the earthquake. Therefore, one can conclude that city infrastructure can efficiently recover if people participate in all phases of the recovery process, even though the received assistance in the emergency OR transitional stages is not adequate. Similar to the second success path of a resilient society (outcome A1), Fakhr Abad presented the second pathway for the success of outcome A2, which revealed that sufficiently managed assistance throughout the project could also lead to a sustainable and resilient built environment.

Amir-al-momenin, Seyyed Taher, and Emamzadeh were the unsuccessful neighborhoods that failed to achieve outcome A2. These neighborhoods all presented one pathway. Sufficient emergency response to basic public needs (condition a1) and sufficient shelters and schools (condition a2) were the only necessary conditions observed in the failure pathway. At the same time, the latter condition also gained high sufficiency (sufficiency = 0.91). The presence of individual conditions such as unreasonable housing design (~a3) and low public engagement (~a5) guaranteed failure, since their sufficiency was high (sufficiency of ~a3 = 0.97 and sufficiency of ~a5 = 0.99).

### 3.3. Resilient Economy (A3)

**Figure 7:**
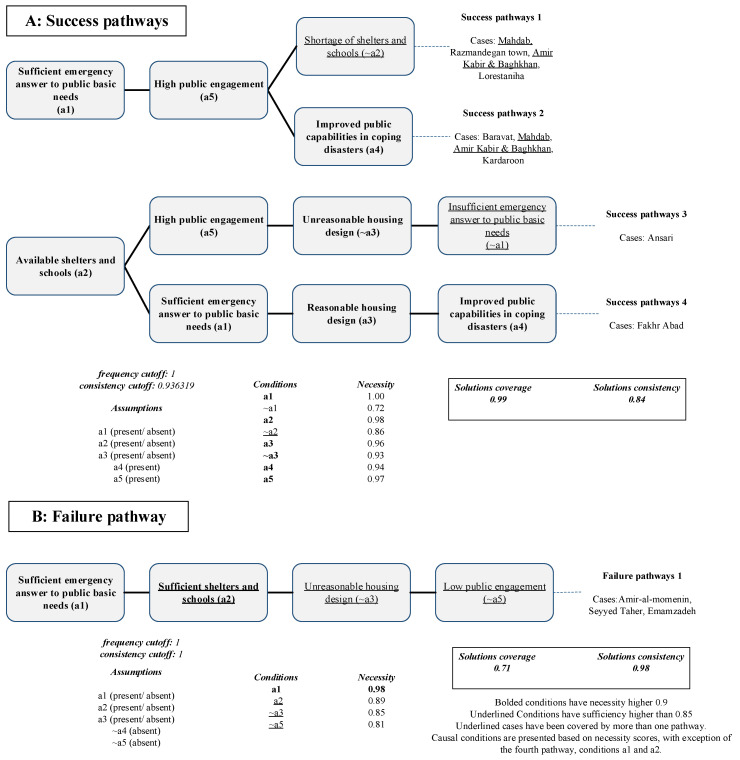
Success and failure pathways for outcome A3.

We identified four possible causal configurations leading to a resilient economy; the overall consistency and coverage scores were 0.84 and 0.99. The pathways for success and failure of a resilient economy are illustrated in Figure 7. A high level of public participation (condition a5) appeared in three of the four pathways, indicating that considering people’s role in economic recovery is crucial. This fact was implied by *Xu*, who explained that the remote resettlement of households can increase livelihood vulnerability [60]. A lack of considering the role of people in long-term recovery was an obstacle in gaining a successfully developed economy after the Wenchuan earthquake, in China, in 2008 [42,61].

Looking at the pathways, emergency response to basic public needs (condition a1), and availability of shelters and schools (a2) appeared across the majority of the neighborhoods and pathways; however, both their presence and absence were observed. Several studies have explored the importance of rescue operations and the availability of shelters with respect to resuming livelihood [59,62]. A poor rescue operation is one of the bottlenecks in recovery projects following disasters [63]. Evidence from real cases and studies has emphasized short temporary accommodations during the emergency and transitional phases due to negative effects on livelihood recovery [3]. However, in our study, the first and third causal configurations imply those conditions (conditions a1 and a2) might interact differently, leading to a resilient economy. While the households in the Amir Kabir and Baghkhan neighborhood had significantly low access to shelters and education and residents lived in the temporary shelters for many months, the rescue team arrived within one day after the earthquake. The residents had sufficient access to sanitation and medical facilities. On the contrary, Ansari’s households faced difficulty getting access to sanitation facilities, and the rescue started much later than in the Amir Kabir and Bghakhan neighborhood. Although the residents were more satisfied with the provided services in the transitional stage, they were less satisfied with the housing design.

Kordaroon, the most successful neighborhood in terms of achieving a resilient economy, presented in the second pathway. According to the interviews, this neighborhood experienced high economic recovery due to high employment rate and increased businesses after the earthquake. This neighborhood showed one of the best improvements in public capabilities to cope with disasters (condition a4), representing adequate financial support and training for the residents. This finding has been supported by a previous study in Bam that reported fast recovery of the businesses that were provided with enough financial assistance [64]. This neighborhood also showed relevantly high access to shelters and education during the transitional phase (condition a2). Located at the city center and the city’s old market, people were highly engaged in the recovery (condition a5).

The negation of outcome A3, illustrating non-resilient recovery, presented one pathway with acceptable coverage and consistency scores. This solution appeared in all the unsuccessful neighborhoods, demonstrating that these neighborhoods that presented limited people participation (~a5) and not climatically and culturally designed houses (~a3) failed to reach a resilient economy, although their emergency and transitional needs were answered (conditions a1 and a2).

### 3.4. Comprehensive and Long-Lasting Success of SNDR Projects Per Community Perspectives (Main Objective)

**Figure 8:**
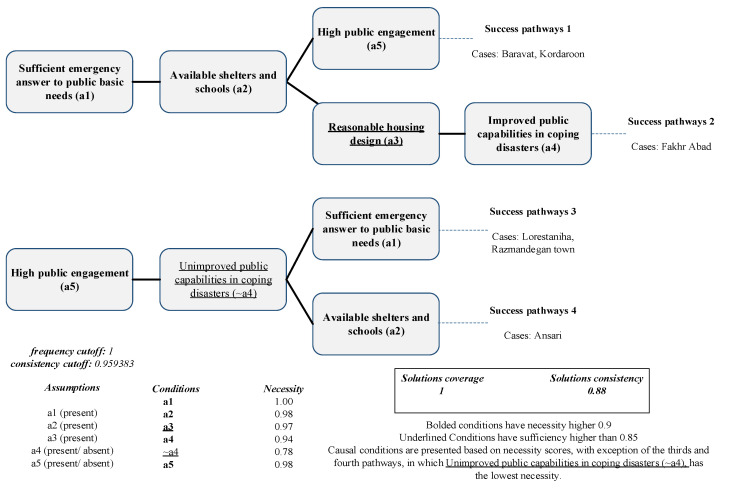
Pathways to the success of the ultimate outcome.

A comprehensive, successful recovery project per the Bam community perspective was conducted by assigning the average score of the final calculated outcomes, and giving twice the weight to resilient economy, per the Delphi result. The fsQCA, finally, suggested three pathways leading to the outcome illustrated in Figure 8. The solution represents all six successful neighborhoods with a coverage score of one and a consistency score of 0.88.

Presented by three pathways and four cases, sufficient emergency response to basic public needs (condition a1) is strongly linked to a successful comprehensive and long-lasting recovery per the residents’ perspectives. The high necessity score of this condition indicated that rescue operations should be promptly conducted to reach this outcome. In addition, high public engagement (condition a5), which was observed in three causal configurations and five neighborhoods, was consistent with previous studies with respect to the role of public engagement in the overall success of a recovery project [10,15]. Neighborhoods such as Ansari were highly engaged in the planning, design, and construction phases. Therefore, the Ansari neighborhood experienced a successful comprehensive and long-lasting recovery per the residents’ perspectives.

The simultaneous presence of sufficient emergency response to basic public needs (condition a1) and high public engagement (condition a5) were observed in the first and third pathways. While Baravat and Kardaroon residents had adequate access to safe shelters and education after the earthquake (condition a2), Lorestaniha and Razmandegan neighborhoods demonstrated low improvement in public capabilities to respond to a disaster (~a4), since most residents were not provided with disaster training. However, all four neighborhoods have successfully achieved the ultimate outcome indicating the necessity of considering the different combinations of the mentioned conditions.

Two of the pathways were separately presented in the Fakhr Abad and Ansari neighborhoods; both neighborhoods included sufficient shelters and schools in the transitional phase (condition a2). The latter condition showed a high necessity score and appeared in three pathways and four neighborhoods. However, the Fakhr Abad neighborhood established a relatively greater improvement in public capabilities to respond to disasters (condition a4) as compared with the Ansari neighborhood. The majority of the residents from the Fakhr Abad neighborhood participated in disaster training, mostly conducted before the earthquake. Moreover, most households received financial assistance lasting one year after the earthquake. On the contrary, the Ansari residents received less disaster training and, although they had access to financial aid, it did not last long enough. However, both communities achieved successful recovery per the residents’ perspectives. Therefore, it is expected that, although formal training was lacking in the Ansari neighborhood, public engagement resulted in informal knowledge transmission, which improved the public’s knowledge about safe construction [23].

Additionally, the Fakhr Abad neighborhood presented the condition, which was both necessary and sufficient. Reasonable housing design (a3), which was only observed in Fakhr Abad was essential to reach this outcome. In addition, climatically and culturally built houses can guarantee achieving success. The findings in the literature also supported this observation. As explained by Yilmaz et al., the government’s efforts to plan standardized houses for all damaged regions may negatively impact house designs and structures [53]. After the Wenchuan earthquake, the unfamiliarity of designers with cultural and local features of the damaged area resulted in inappropriate designs and dissatisfaction among residents [11].

A close look at Table 5, obtained from the study’s truth table, demonstrates that a minor difference in the causal configuration leads to a major change in the results. These Insufficient but Necessary components of Unnecessary but Sufficient causes are called INUS causes [20]. Considering unique circumstances, the presence or absence of one condition can make a significant difference in the pathways. A comparison between the following casual configurations revealed that people’s participation is a critical condition whose absence results in failure. While the neighborhoods showed similarity in the combination of the conditions, except for condition a5, the Kardaroon and Baravat neighborhoods successfully reached the ultimate outcome. In contrast, low public engagement (condition a5) in the Emamzade, Amir-al-momenin, and Seyyed Taher neighborhoods, resulted in failure to achieve the ultimate outcome.

The solution for non-successful recovery showing two possible pathways covering all five unsuccessful neighborhoods is illustrated in Table 5. The solution’s coverage is 0.8, and its coverage is perfect. Improved public capabilities in coping with disasters (condition a4) is necessary for the outcome’s failure, although it is not sufficient. Unreasonable housing design (~a3), low public engagement (~a5), and shortage of shelters and schools (~a2) are highly sufficient, although their necessity score is lower than 0.9 (sufficiency of ~a3 = 0.97, sufficiency of ~a5 = 0.98 and, sufficiency of ~a2 = 0.98).

**Figure 9:**
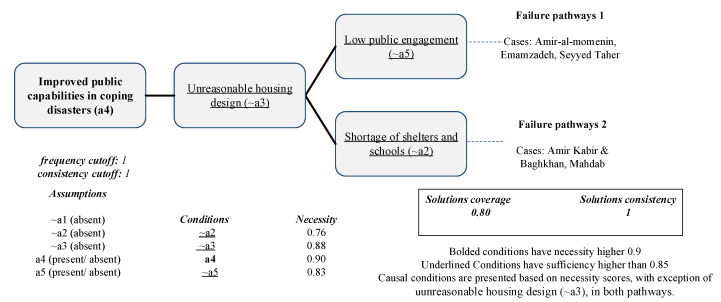
Pathways to failure of the ultimate outcome.

The failure pathways presented in Figure 9 both share improved public capabilities in coping with disasters (condition a4) and unreasonable housing design (~a3). Amir-al momenin, Seyyed Taher, and Emamzadeh can be observed in the first failure pathway, and the second solution presented Amir Kabir and Baghkhan and Mahdab. Seyyed Taher presented the lowest score for reasonable housing design (~a3), and the public was not actively engaged in that neighborhood. More than half the respondents in Seyyed Taher preferred living in their old houses, and all of them had made modifications in their housing design. Moreover, public engagement in all recovery phases was significantly low (~a5). In addition, the children did not have access to education during the transitional phase in the Mahdab neighborhood, which had the lowest score for shelter and education availability (~a2). The residents also lived in transitional houses for years after the earthquake. The combination of the mentioned shortcomings has resulted in those neighborhoods failing to achieve successful recovery per the residents’ perspectives; however, low public engagement may have had a greater impact on the failure due to its higher necessity score.

## 4. Conclusions

This study demonstrates how communities can successfully recover after such a severe earthquake. Although the disaster recovery evaluation was mostly conducted considering separate factors, this study attempted to evaluate how combinations of the elements might lead to the success or failure of a recovery project. While it is expected that the simultaneous presence of all five conditions might lead to a successful SNDR project, the results have demonstrated how combinations of conditions can change the project’s consequences. The original contributions of this research are outlined below.

- The research findings illustrate the determinant role of public engagement and emergency response in the recovery process. Public engagement played a vital role in achieving a resilient society, sustainable and resilient built environment, resilient economy, and long-lasting recovery success per community perspectives. However, the emergency response showed a strong link with successfully achieving a resilient society and long-lasting recovery per community perspectives. Moreover, the analysis of the success and failure pathways of successful long-lasting recovery per the residents’ view illustrated the importance of public engagement. Communities provided with sufficient emergency and transitional response did not experience comprehensive recovery years after the disaster unless people actively participated in the recovery process. This finding emphasizes the need for active engagement of all people in all phases of recovery. However, as discussed by some researchers, community engagement in the recovery process should be conducted under the clear guidance of governments to balance the impact on results [6,18].
- Individual factors have an impact on the success of a project. However, a combination of sufficient emergency response (rescue and health service), adequate response through to the transitional phase (shelter availability and living duration in permanent houses and shelters), climatically and culturally designed houses, and enhanced public strength through financial aid and disaster training, could achieve recovery success. The Fakhr Abad neighborhood, presenting the mentioned pathway, achieved full economic, social, and built environment recovery and ranked as a successful project per the residents’ perspectives. Therefore, it is necessary to provide sufficient answers to all the recovery phases, from rescue operations to public capability enhancement.
- One of the most interesting findings of this study is the significant role of housing design. Cultural housing design has been recognized as a crucial parameter affecting the satisfaction of residents [47]. Since shared living and working places are commonly seen in rural neighborhoods, it is necessary to consider the needs of people in housing design to avoid affecting their livelihoods [3]. The results of this study reveal that reasonable housing design is a necessary condition to achieve all four outcomes. Moreover, on the other hand, the climatically and culturally designed houses guaranteed recovery success based on Bam’s neighborhoods. On the other hand, the recovery project failed to reach all the outcomes in neighborhoods with inappropriately designed houses.

Since the Bam earthquake located in a historical city with significant characteristics was the case study selected for this research, it was not expected that the same results would be obtained from different disaster recovery projects. Further research is needed to identify the success pathways for various disasters in other countries. Finally, the goal of this study was to identify the ways that led to successful recovery in Bam based on public perspective. Since SNDR projects involve many stakeholders, it is crucial to structure further studies to analyze the recovery pathways following various beneficiaries’ points of view.

## Figures and Tables

**Table 1:** Comprehensive and long-lasting success dimension of SNDR projects based on the community perspective.

No	Outcome	Delphi Mean Score (from 5)	Indices	Source
**A1**	Resilient Society	3.8	Social connections	[10,14,15,26]
			Psychological support (suicide, mental disorders report	[13,32,33]
			Safety (criminal behavior)	[14,27,34]
			Life satisfaction and satisfaction of recovery process	[35,36,37]
**A2**	Sustainable and resilient built environment	3.66	Population per capita in houses	[13,27,29]
			Hygiene water accessibility	[19,28,31]
			Electricity accessibility	[19,28,31]
			Road improvement	Case knowledge
			Number of city facilities (schools, hospitals)	Case knowledge
**A3**	Resilient economy	4.3	Number of new businesses initiation or restoration of old ones	[32,38,39,40]
			Employment growth rate	[14,38]
			Household income growth rate	[19], Case knowledge

**Table 2:** Hypothesized causal conditions that influence the success dimensions of SNDR projects based on the community perspective.

No	Conditions	Indices	Sources
a1	Sufficient emergency answer to basic public needs *	Sanitation level	[13,37,41]
		Starting time for rescue and site investigation	[22,42]
a2	Availability of shelter and schools *	Shelter availability	[43,44,45]
		School availability	[15,46]
a3	Reasonable housing design **	Consideration of local culture and community needs	[29,31,47]
		Consideration of local climate	[32,48]
a4	Improvement of public capabilities ***	Disaster and safe construction training	[3,15,49,50]
		Livelihood development	[51,52]
a5	Community engagement level ***	Decision-making	[52,53]
		Planning	[23,35,54]
		Construction	[23,55]

NOTE: The conditions marked by * indicate the emergency and transitional recovery activities, while ** and *** stand for mid-term and long-term recovery steps, respectively.

**Table 3:** Examples of variable calibration.

A2 (Sustainable and Resilient Built Environment)				
**Average of B5–B9, with Twice Weight Given to B6 and B7**				
**B5, Population Per Capita in Houses**		**B6, Hygiene Water Accessibility**	**B7, Electricity Accessibility**	
(B5-1) Changes of the available housing area for each person before and after the earthquake: *0 Decrease in the available housing area for each person* *1 Increase in the available housing area for each person*		(B6-1) Resuming time of the water availability in the neighborhood: *0 More than two years* *0.4 One–two years* *0.8 6 months to one year* *1 Less than 6 months*	(B7-1) Resuming time of the electricity availability in the neighborhood: *0 More than two years* *0.4 One–two years* *0.8 6 months to one year* *1 Less than 6 months*	
B5 = sum of scores of B5-1		B6 = sum of scores of B6-1	B7 = sum of scores of B7-1	
**B8, Road Improvement**			**B9, Education and Hospital Improvement**	
(B8-1) Resuming time of the roads in the neighborhood: *0 Some roads have not been finished yet.* *0.2 More than three years 0.4 Two-three years 0.7 One–two years* *1 Less than one year*	(B8-2) Road quality: *0 Extremely worse now* *0.2 Worse now* *0.4 No change* *0.7 Better now* *1 Extremely better now*	(B8-3) Accessibility to the Province center and nearby cities: *0 It is much difficult now.* *0.5 It has not changed.* *1 It is much easier now.*	(B9-1) Education quality: *0 Extremely worse now* *0.2 Worse now* *0.4 No change* *0.7 Better now* *1 Extremely better now*	(B9-2) Medical service quality: *0 Extremely worse now* *0.2 Worse now* *0.4 No change* *0.7 Better now* *1 Extremely better now*
B8 = minimum of B8-1, B8-2, B8-3			B9 = average of B9-1 and B9-2	

**Table 4:** Truth table; case scores.

Case Num	Case Name	a1	a2	a3	a4	a5	A1	A2	A3	ULTIMATE
***1***	*Amir Kabir & Baghkhan*	0.820	0.436	0.49	0.729	0.522	0.43	0.44	0.42	0.428
***2***	*Mahdab*	0.724	0.286	0.426	0.528	0.556	0.37	0.42	0.38	0.386
***3***	*Fakhr Abad*	0.750	0.642	0.509	0.881	0.411	0.61	0.44	0.47	0.498
***4***	*Razmandegan town*	0.716	0.411	0.871	0.498	0.556	0.44	0.47	0.38	0.418
***5***	*Emamzadeh*	0.814	0.665	0.474	0.539	0.444	0.47	0.43	0.42	0.436
***6***	*Amir-al-momemnin*	0.593	0.805	0.398	0.639	0.389	0.63	0.28	0.34	0.396
***7***	*Lorestaniha*	0.575	0.480	0.432	0.432	0.647	0.49	0.47	0.5	0.488
***8***	*Koradoon*	0.667	0.676	0.492	0.799	0.520	0.34	0.43	0.59	0.488
***9***	*Ansari*	0.477	0.580	0.409	0.250	0.791	0.52	0.54	0.46	0.494
***10***	*Baravat*	0.650	0.640	0.492	0.796	0.556	0.44	0.34	0.39	0.390
***11***	*Seyyed Taher*	0.651	0.589	0.444	0.527	0.417	0.32	0.46	0.41	0.400

**Table 5:** INUS causes of the ultimate outcome. The absence of condition a5 resulted in recovery failure.

Neighbrhood Name/Condition	a1	a2	a3	a4	a5	Ultimate Outcome
Kardaroon, Baravet	1	1	0	1	1	1
Emamzageh, Amir-al-momenin, Seyyed Taher	1	1	0	1	0	0

## Data Availability

All data generated or analyzed during this study are included in this published article.

## Appendix A. Calibration Rubrics

**Table A1:** Conditions’ calibration rubrics.

Conditions’ Calibration Rubrics					
a1 (Sufficient emergency answer to basic public needs)	Average of b1and b2, with twice weight given to b2				
	b1, Sanitation level			b2, Rescue and site investigation	
	**(b1-1) Water availability in the shelters:** [0] No access to public toilets and difficulty accessing water [0.33] Access to public toilets but difficulty accessing water [0.67] No access to public toilets, but access to water [1] Access to both public toilets and water	**(b1-2) Medical service availability in the shelters:** [0] No access [0.5] Sometimes available [1] Always available		**(b2-1) Time of first rescue team arrival:** [1] Next day after the earthquake [0.8] Within three days after the earthquake [0.4] 4-7 days after the earthquake [0] One week after the earthquake	
	b1 = average of b1-1 and b1-2, with twice weight given to b1-1			B2 = sum of scores for b2-1	
A2 (Availability of shelters and schools)	Average of b3 and b4, with twice weight given to b3				
	b3, Education availability	b4, Shelter availability			
	**(b3-1) Teachers’s availability after the earthquake:** 1 Volunteer [1] Governmental organization (ministry of education) [1] Non-governmental organization [0] The children did not have teachers	**(b4-1) Waiting time to find emergency shelter:** [0] More than one month [0.2] 2–4 weeks [0.4] 1–2 weeks [0.7] One week [1] Provided by a shelter just after the earthquake		**(b4-2) Duration of living in emergency shelters:** [0] More than 2 years [0.2] 1–2 years [0.4] 6 months to 1 year [0.7] 2–6 months [1] Less than 2 months	**(b4-3) Duration of living in transitional homes:** [0] More than 2 years [0.2] 1–2 years [0.4] 6 months to 1 year [0.7] 2–6 months [1] Less than 2 months
	b3 = sum of scores for b3-1	b3 = average of 4-1, b4-2, b4-3			
a3 (Reasonable housing design)	Average of b5 and b6, with twice weight given to b5				
	b5, housing based on local culture and needs			b6, Climate consideration in housing design	
	**(b5-1) Living experience, preference of old or new house:** [0] The old house [0.5] Both are the same. [1] New house	**(b5-2)** **House layout modifications:** Added storing/business place [0] Added rooms [0] Changed the washroom’s place [0] Extension of kitchen or yard [0] Other changes [1] No change		**(b6-1) Indoor thermal comfort, winter:** [0] Freezing, intolerable [0.5] Moderate, tolerable [1] Warm, desirable	**(b6-2) Indoor thermal comfort, summer:** [0] Scorching, intolerable 0 [0.5] Moderate, tolerable [1] Cool, desirable
	b5 = maximum of scores for b5-1 and b5-2			b6 = average of b6-1 and b6-2	
a4 (Improvement of public capabilities)	Minimum of b7 and b8				
	b7 Community training			b8, Livelihood development	
	**(b7-1) Training availability:** [1] Emergency aids [1] Rescuing and self-protection [1] Safe construction [1] Others [0] No	**(b7-2) Training time:** [0] No training [0.5] After the earthquake [1] Before the earthquake		**(b8-1) Received financial aids:** [1] House rebuild/repair grant [1] Bank loans for housing [1] Subsidy/grant for resuming/starting a business [1] Bank loans for resuming/starting a business [1] Monthly stipend [1] Other [0] Nothing	**(b8-2) Duration of financial aids:** [0] No financial aids [0.33] Less than 6 months [0.67] 6 months to 1 year [1] Until the end of housing reconstruction or resuming/starting a business
	b7 = average of b7-1 and b7-2			b8 = average of b8-1 and b8-2	
a5 (Community engagement level)	Average of b9 and b10, with twice weight given to b10				
	b9, Public participation in decision-making		b10, Public participation in construction		
	**(b9-1) Public participation in site selection:** [0] Relocated to the new site [1] Settled in the old site	**(b9-2) Keeping public informed:** [1] By Shora (community centers) [1] By local news [1] By Setads [1] By volunteers/non-governmental organizations [1] By neighbors/friends [1] Other [0] No shared information	**(b10-1) Public participation in planning:** [0] Choosing one of the portfolio plans for house layout [1] Participation in housing planning with the consultant’s assistance	**(b10-2) Public participation in reconstruction:** [0] Choosing the contractor and supervision on his work [1] Self-construction as a contractor	**(b10-3) Public participation in procurement:** [0] Contractor in charge of purchasing materials [1] Householder in charge of purchasing materials
	b9 = average of scores for b9-1 and b9-2		b10 = average of b10-1, b10-2, b10-3		

**Table A2:** Outcomes’ calibration rubrics.

Outcomes’ Calibration Rubrics						
A1 (Resilient Society)	Average of B1, B2, B3, and B4					
	B1, Social connection	B2, Psychological support	B3, safety and security	B4, Life satisfaction		
	**(B1-1) Living with the same family and neighbors after the earthquake:** [0] Loss of family members due to death or immigration (earthquake) [0.5] There are new members in the community or family. [1] All the social connections are the same as before the earthquake	**(B2-1) Availability of psychological consultation after the earthquake:** [1] By volunteers [1] By Red Cresent of Iran [1] By friend/relatives [1] By non-governmental organizations [0] No consultation	**(B3-1) Experience of robbery/crime after the earthquake:** [0] Repeatedly [0.2] Often [0.4] Sometimes [0.7] Few times [1] Rarely	**(B4-1) Living experience, preference of old or new house:** [0] The old house 0 [0.5] Both are the same. [1] New house	**(B4-2) City’s major changes after the earthquake:** [0] Decline in industry [1] Boomed industry [0] Decline in agriculture [1] Boomed agriculture [0] Decline in tourism [1] Boomed tourism [0] Polluted environment [1] Cleaner environment [0] Worse transportation system [1] Better transportation system [0] Worse education, health system [1] Better education, health system [0] Unsafe and uncomfortable houses [1] Safer and better houses	
	B1 = sum of scores of B1-1	B2 = sum of scores of B2-1	B3 = sum of score of B3-1	B4 = maximize of B4-1 and B4-2		
A2 (Sustainable and resilient built environment)	Average of B5- B9, with twice weight given to B6 and B7					
	B5, Population per capita in houses		B6, Hygiene water accessibility		B7, Electricity accessibility	
	**(B5-1) Changes of the available housing area for each person before and after the earthquake:** [0] Decrease in the available housing area for each person [1] Increase in the available housing area for each person		**(B6-1) Resuming time of the water availability in the neighborhood:** [0] More than two years [0.4] One–two years [0.8] 6 months to one year [1] Less than 6 months		**(B7-1) Resuming time of the electricity availability in the neighborhood:** [0] More than two years [0.4] One–two years [0.8] 6 months to one year [1] Less than 6 months	
	B5 = sum of scores of B5-1		B6 = sum of scores of B6-1		B7 = sum of scores of B7-1	
	B8, Road improvement			B9, Education and hospital improvement		
	**(B8-1) Resuming time of the roads in the neighborhood:** [0] Some roads have not been finished yet. [0.2] More than three years [0.4] Two-three years [0.7] One–two years [1] Less than one year	**(B8-2) Road quality:** [0] Extremely worse now [0.2] Worse now [0.4] No change [0.7] Better now [1] Extremely better now	**(B8-3) Accessibility to the Province center and nearby cities:** [0] It is much difficult now. [0.5] It has not changed. [1] It is much easier now.	**(B9-1) Education quality:** [0] Extremely worse now [0.2] Worse now [0.4] No change [0.7] Better now [1] Extremely better now	**(B9-2) Medical service quality:** [0] Extremely worse now [0.2] Worse now [0.4] No change [0.7] Better now [1] Extremely better now	
	B8 = minimum of B8-1, B8-2, B8-3			B9 = average of B9-1 and B9-2		
A3 (Resilient economy)	Average of B10, B11 and B12, with twice weight given to B12					
	B10, Business recovery			B11, Employment rate		B12, Household income
	**(B10-1) Old businesses recovery:** [0] No re-operation of the old businesses [0.5] Partial recovery of the old businesses [1] Full recovery of the old businesses	**(B10-2) Business growth rate (before and after the earthquake):** [0] Declined [0.5] No change [1] Increase	**(B20-3) Job stability in the city:** [0] People with changed career [1] People with the same career	**(B11-1) Employment status:** [0] Employee [1] Business owner	**(B11-2) Working place during reconstruction:** [0] Other places [1] Bam	**(B12-2) Household income change before and after the earthquake:** [0] Better income before the earthquake [0.5] No change in income [1] Better income after the earthquake
	B10 = average of B10-1, B10-2, B10-3			B11 = average of B11-1 and B11-2		B12 = sum of scores of B12-1

## Appendix B. Data Analysis through fsQCA Software

### Appendix B.1. Outcome A1

1. The calculation started by computing the necessity and sufficiency of each condition to reach the outcome.

**Table A3:** Necessity and sufficiency analysis of conditions (outcome A1).

Conditions	Necessity	Sufficiency
a1	0.984573	0.669356
~a2	0.798814	0.843841
a2	0.977057	0.795491
a3	0.901899	0.835624
a4	0.935918	0.715020
~a4	0.742490	0.857371
a5	0.907437	0.789809

As no clear theoretical background between the emergency response to people’s basic needs and the resilience of a society has been found, the model has considered this condition’s absence and presence (a1). Therefore, using ~a4 as the prime implicant, the intermediate solution has been formulated as below.

***Assumptions:***

a1 (present/absent)
a2 (present)
a3 (present)
a4 (present)
a5 (present)
*frequency cutoff: 1*
*consistency cutoff: 0. 915578*

**Table A4:** Success pathways (outcome A1)- The mark * resembles “and”.

Solutions	Raw Coverage	Consistency
a1*~a2*a5	0.798814	0.886987
a2*~a4*a5	0.714822	0.9584
a1*a2*a3*a4	0.847035	0.926903

*solution coverage: 0. 93419*
*solution consistency: 0.852787*

This solution shows high consistency and coverage, and the pathways make sense according to both theory and case knowledge. The final pathways of the resilient community can then be seen within the following pathways:

i. Suitable emergency response to basic public needs (a1) AND high community engagement (a5) AND insufficient shelter and school (~a2)

OR

ii. Suitable emergency response to basic public needs (a1) AND Sufficient shelter and school (a2) AND improved public capabilities (a4) AND resilient housing design (a3)

OR

iii. Sufficient shelter and school (a2) AND high community engagement (a5) AND unimproved public capabilities (~a4).
2. The negate of the conditions that have been considered in the resilient society formed the necessity and sufficiency of each condition impacting the non-resilient community.

**Table A5:** Necessity and sufficiency analysis of conditions (outcome ~A1).

Conditions	Necessity	Sufficiency
~a1	0.586306	0.978108
a1	0.994616	0.794944
a2	0.874074	0.836071
~a2	0.786339	0.975783
~a3	0.849092	0.910518
~a4	0.682705	0.926061
~a5	0.794583	0.909844
a5	0.896198	0.917025
a3	0.841353	0.916438

The model has considered both the absence or presence of a1 while all the rest of the conditions were absent. Additionally, the researcher has selected a1*a2*~a3 as the prime implicant.

***Assumptions:***

a1 (present/absent)
~a2 (absent)
~a3 (absent)
~a4 (absent)
~a5 (absent)
*frequency cutoff: 1*
*consistency cutoff: 0.927582*

**Table A6:** Failure pathways (outcome A1)- The mark * resembles “and”.

Solutions	Raw Coverage	Consistency
a1*a2*~a3	0.785522	0.93507

*solution coverage: 0.785522*
*solution consistency: 0.93507*

The pathways for the non-resilient society are presented below:

i. Sufficient emergency answer to basic public needs (a1) AND Adequate shelters and schools (a2) AND Unreasonable housing design (~a3)

### Appendix B.2. Outcome A2

1. The results of the necessity and sufficiency analysis for outcome A2 are shown below. While the conditions’ necessity has gained high scores, the sufficiency values are lower.

As no clear theoretical background between this outcome and the prompt answer to the emergency needs and availability of the shelters and schools have been found, the model has considered both the absence and presence of a1 and a2. However, the rest of the conditions have been assumed to be present. Using the prime implicant of a2 * a4 * a5, the intermediate solution is formulated below.

**Table A7:** Necessity and sufficiency analysis of conditions (outcome A2).

Conditions	Necessity	Sufficiency
a1	0.986856	0.625924
~a1	0.724401	0.959023
a2	0.957176	0.727053
~a2	0.887640	0.874113
a3	0.960356	0.830126
a4	0.930040	0.662889
a5	0.984736	0.799621

***Assumptions:***

a1(present/absent)
a2 (present/absent)
a3 (present)
a4 (present)
a5 (present)
*frequency cutoff: 1*
*consistency cutoff: 0.912352*

**Table A8:** Success pathways (outcome A2)- The mark * resembles “and”.

Solutions	Raw Coverage	Consistency
a1*~a2*a5	0.886229	0.917928
~a1*a2*a5	0.725847	0.966432
a1*a2*a3*a4	0.885381	0.903763
a2*a4*a5	0.873517	0.905756

*solution coverage: 0.993008*
*solution consistency: 0.840265*

The final pathways of the sustainable and resilient built environment can then be seen within the following pathways:

i. High public engagement (a5) AND Sufficient shelters and schools (a2) AND Insufficient emergency answer to basic public needs (a4)

OR

ii. High public engagement (a5) AND Sufficient shelters and schools (a2) AND Unimproved public capabilities in coping with disasters (~a1)

OR

iii. Sufficient emergency answer to basic public needs (a1) AND Reasonable housing design (a3) AND Sufficient shelters and schools (a2) AND Improved public capabilities in coping with disasters (a4)

OR

iv. Sufficient emergency answer to basic public needs (a1) AND High public engagement (a5) AND Insufficient shelters and schools (~a2).

2. The necessity and sufficiency of all the negation of the conditions that appeared in the last section have been calculated for the negation of outcome A2.

**Table A9:** Necessity and sufficiency analysis of conditions (outcome ~A2).

Conditions	Necessity	Sufficiency
~a1	0.557218	0.982599
a1	0.976763	0.825198
a2	0.904027	0.914654
~a2	0.730224	0.957829
a3	0.814261	0.937512
~a3	0.852459	0.966264
~a5	0.814738	0.986130

The negation model has considered the absence and presence of a1 and a2, while all the rest of the conditions were absent (contrary to the outcome A2). The below assumptions have been considered to form the intermediate solution presented below.

***Assumptions:***

a1 (present/absent)
a2 (present/absent)
~a3 (absent)
~a4 (absent)
~a5 (absent)
*frequency cutoff: 1*
*consistency cutoff: 1*

**Table A10:** Failure pathways (outcome A2)- The mark * resembles “and”.

Solutions	Raw Coverage	Consistency
a1*a2*~a3*~a5	0.811873	0.986081

*solution coverage: 0.71*
*solution consistency: 1*

This solution shows high consistency and coverage, and the pathways make sense according to both theory and case knowledge. Furthermore, the pathways for the non-sustainable and resilient built environment are presented below:

i. Sufficient emergency answer to basic public needs (a1) AND Adequate shelters and schools (a2) AND unreasonable housing design (~a3) AND low public engagement (~a5).

### Appendix B.3. Outcome A3

1. To start modeling the pathways to outcome A3, the conditions’ necessity and sufficiency values for the outcome have been computed as follows.

**Table A11:** Necessity and sufficiency analysis of conditions (outcome A3).

Conditions	Necessity	Sufficiency
a1	1.000000	0.639505
~a1	0.721404	0.962953
a2	0.977502	0.748631
~a2	0.857023	0.850939
a3	0.955214	0.832509
~a3	0.929563	0.797583
a4	0.942809	0.677546
~a4	0.774160	0.840940
a5	0.972456	0.796178

While theoretical links between the outcome and presence of conditions, a1, a2, and a3 have not been found, the presence and absence of those conditions have been assumed. Using the prime implicant of a2*a3 and a1*a2*a4*a5, the intermediate solution is formulated below.

***Assumptions:***

a1 (present/absent)
a2 (present/absent)
a3 (present/absent)
a4 (present)
a5 (present)
*frequency cutoff: 1*
*consistency cutoff: 0.935643*

**Table A12:** Success pathways (outcome A3)-.

Solutions	Raw Coverage	Consistency
a1*~a2*a5	0.857563	0.895765
~a1*a2*~a3*a5	0.688655	0.966962
a1*a2*a3*a4	0.901261	0.927768
a1*a4*a5	0.914496	0.876384

*solution coverage: 0.985294*
*solution consistency: 0.840803*
The mark * resembles “and”.

In order to simplify the solutions, the researcher computed subset/superset analysis of each solution and tried to find the subsets with higher coverage within the same range of the consistency score (higher than 0.85). However, as most of the factors have appeared in several pathways, the researcher did not change or reduce the conditions of the analysis. The final pathways of the resilient economy can then be seen within the following pathways:

i. Sufficient emergency answer to basic public needs (a1) AND High public engagement (a5) AND Inadequate shelters and schools (~a2)

OR

ii. Sufficient emergency answer to basic public needs (a1) AND High public engagement (a5) AND Improved public capabilities in coping with disasters (a4)

OR

iii. Sufficient emergency answer to basic public needs (a1) AND Available shelters and schools (a2) AND Reasonable housing design (a3) AND Improved public capabilities in coping with disasters (a4)

OR

iv. Available shelters and schools (a2) AND High public engagement (a5) AND Unreasonable housing design (~a3) AND Insufficient emergency answer to basic public needs (~a1).

2. To model the negation on outcome A3, each condition’s necessity and sufficiency values have been calculated first.

**Table A13:** Necessity and sufficiency analysis of conditions (outcome ~A3).

Conditions	Necessity	Sufficiency
~a1	0.570628	1.000000
a1	0.978860	0.821837
a2	0.885650	0.890499
~a2	0.750000	0.977662
a3	0.820307	0.938611
~a3	0.853619	0.961573
~a4	0.658232	0.937928
~a5	0.810378	0.974764
a4	0.886931	0.836809
a5	0.849615	0.913238

The assumptions for the presence or absence of the modeling followed the same rules as the last phase, while ~a4 is the selected prime implicant. The intermediate solution is shown as follows:

***Assumptions:***

a1 (present/absent)
a2 (present/absent)
a3 (present/absent)
~a4 (absent)
~a5 (absent)
*frequency cutoff: 1*
*consistency cutoff: 0.984354*

**Table A14:** Failure pathways (outcome A3)- The mark * resembles “and.”.

Solutions	Raw Coverage	Consistency
a1*a2*~a3*~a5	0.710417	0.984455

*solution coverage: 0.710417*
*solution consistency: 0.984455*

The final pathways of the non-resilient economy can then be seen within the following pathways:

i. Sufficient emergency answer to basic public needs (a1) AND Adequate shelters and schools (a2) AND Unreasonable housing design (~a3) Low public engagement (~a5).

#### Comprehensive and Long-Lasting Success of SNDR Projects (Communities Perspective)

1. The average of the scores of the outcomes, A1, A2, and A3, given twice weight to A3 following the mean scores by the Delphi result, has formed the values for the outcomes. Similar to the last outcomes, necessity and sufficiency analysis has been calculated first.

**Table A15:** Necessity and sufficiency analysis of conditions (ultimate goal).

**Conditions**	**Necessity**	**Sufficiency**
a1	0.996474	0.646094
a2	0.976151	0.757971
a3	0.970759	0.857797
a4	0.937785	0.683288
~ a4	0.783492	0.862163
a5	0.980506	0.813909
~a5	0.911240	0.846465

The absence/presence of conditions a4 and a5 have been assumed, as these factors may not tangibly affect the outcome compared to the rest of the three conditions. The intermediate solution has been formulated as below.

***Assumptions:***

a1 (present)
a2 (present)
a3 (present)
a4 (present/absent)
a5 (present/absent)
*frequency cutoff: 1*
*consistency cutoff: 0.959383*

**Table A16:** Success pathways (ultimate goal)- The mark * resembles “and.”.

Solutions	Raw Coverage	Consistency
a1*~a4*a5	0.779967	0.931863
a2*~a4*a5	0.759643	0.970588
a1*a2*a5	0.953132	0.952144
a1*a2*a3*a4	0.91559	0.954801

*solution coverage: 1*
*solution consistency: 0.883312*

Following the last table, the final pathways of the successful SNDR project from the community perspective can then be seen within the following pathways:

i. Suitable emergency response to basic public needs (a1) AND Sufficient shelter and school (a2) AND high community engagement (a5)

OR

ii. Suitable emergency response to basic public needs (a1) AND Sufficient shelter and school (a2) AND Resealable housing design (a3) AND Improved public capabilities (a4)

OR

iii. High community engagement (a5) AND Unimproved public capabilities (~a4) AND Suitable emergency response to basic public needs (a1)

OR

iv. High community engagement (a5) AND Unimproved public capabilities (~a4) AND Sufficient shelter and school (a2).

2. The second step has been initiated by computing the necessity and sufficiency analysis for each condition (negate the last phase).

**Table A17:** Necessity and sufficiency analysis of conditions (~ultimate goal).

Conditions	Necessity	Sufficiency
a1	0.993525	0.825333
~a1	0.573972	0.995229
~a2	0.756717	0.975992
~a3	0.874393	0.974562
a4	0.902234	0.842248
~ a4	0.660732	0.931538
a5	0.870994	0.926321
~a5	0.825024	0.981892

The model has considered both the absence and presence of a4 and a5, while the rest of the conditions were absent. Subsequently, the intermediate solution has been formulated as below.

***Assumptions:***

~a1 (absent)
~a2 (absent)
~a3 (absent)
a4 (present/absent)
a5 (present/absent)
*frequency cutoff: 1*
*consistency cutoff: 1*

**Table A18:** Failure pathways (ultimate goal)- The mark * resembles “and.”.

Solutions	Raw Coverage	Consistency
~a3*a4*~a5	0.758012	1
~a2*~a3*a4	0.619294	1

*solution coverage: 0.795241*
*solution consistency: 1*

The final pathways of the non-resilient economy can then be seen within the following pathways:

i. Improved public capabilities (a4) AND Unreasonable housing design (~a3) AND low community engagement (~a5)

OR

ii. Improved public capabilities (a4) AND Unreasonable housing design (~a3) AND Insufficient shelters and schools (~a2).
